# SWAMPy: simulating SARS-CoV-2 wastewater amplicon metagenomes

**DOI:** 10.1093/bioinformatics/btae532

**Published:** 2024-09-03

**Authors:** William Boulton, Fatma Rabia Fidan, Hubert Denise, Nicola De Maio, Nick Goldman

**Affiliations:** European Molecular Biology Laboratory, European Bioinformatics Institute (EMBL-EBI), Hinxton, Cambs CB10 1SD, United Kingdom; Department of Computing Sciences, University of East Anglia, Norwich, Norfolk NR4 7TJ, United Kingdom; European Molecular Biology Laboratory, European Bioinformatics Institute (EMBL-EBI), Hinxton, Cambs CB10 1SD, United Kingdom; Department of Biological Sciences, Middle East Technical University, Ankara 06800, Turkey; Cancer Dynamics Laboratory, Francis Crick Institute, London NW1 1AT, United Kingdom; Department of Health and Social Care, UK Health Security Agency, London SW1P 3HX, United Kingdom; European Molecular Biology Laboratory, European Bioinformatics Institute (EMBL-EBI), Hinxton, Cambs CB10 1SD, United Kingdom; European Molecular Biology Laboratory, European Bioinformatics Institute (EMBL-EBI), Hinxton, Cambs CB10 1SD, United Kingdom

## Abstract

**Motivation:**

Tracking SARS-CoV-2 variants through genomic sequencing has been an important part of the global response to the pandemic and remains a useful tool for surveillance of the virus. As well as whole-genome sequencing of clinical samples, this surveillance effort has been aided by amplicon sequencing of wastewater samples, which proved effective in real case studies. Because of its relevance to public healthcare decisions, testing and benchmarking wastewater sequencing analysis methods is also crucial, which necessitates a simulator. Although metagenomic simulators exist, none is fit for the purpose of simulating the metagenomes produced through amplicon sequencing of wastewater.

**Results:**

Our new simulation tool, SWAMPy (**S**imulating SARS-CoV-2 **W**astewater **A**mplicon **M**etagenomes with **Py**thon), is intended to provide realistic simulated SARS-CoV-2 wastewater sequencing datasets with which other programs that rely on this type of data can be evaluated and improved. Our tool is suitable for simulating Illumina short-read RT–PCR amplified metagenomes.

**Availability and implementation:**

The code for this project is available at https://github.com/goldman-gp-ebi/SWAMPy. It can be installed on any Unix-based operating system and is available under the GPL-v3 license.

## 1 Introduction

Wastewater sequencing has proven useful in the genomic surveillance of SARS-CoV-2 and can provide a less biased picture of the variants circulating in a population than clinical surveillance (Brown *et al.* 2021). Amplicon sequencing is the preferred method for this purpose since it is efficient in terms of cost, labor, and time, and is well-suited for heavily contaminated samples—as may be found with biological samples collected for SARS-CoV-2 sequencing—thanks to its targeted nature (Hourdel *et al.* 2020). Such sequencing has typically been done via multiplex PCR using a pre-defined primer set with paired-end reads generated by an Illumina device (Brown *et al.* 2021).

A number of methods and software tools for wastewater SARS-CoV-2 sequencing data analysis are available such as SAM Refiner (Gregory *et al.* 2021), COJAC (Jahn *et al.* 2022), LCS (Valieris *et al.* 2022) and Freyja (Karthikeyan *et al.* 2022). Evaluating the effectiveness of new methods on *in vivo* or *in vitro* samples is often difficult or impossible, e.g. because of the lack of availability of a wide range of real or synthetic samples and the costs of repeated experiments (Angly *et al.* 2012). However, simulated datasets can provide an efficient way of benchmarking the performance of new methods (Escalona *et al.* 2016).

There is a specific set of features characteristic of data coming from wastewater amplicon sequencing. For example, it has been shown that there is a high variation in amplification across different amplicons of a given primer set, resulting in a variation in read depth across the genome (Hourdel *et al.* 2020, Brown *et al.* 2021). Moreover, wastewater data are expected to represent a mixture of different SARS-CoV-2 variants since the biological matter in the sample comes from multiple people, and will carry RNA degradation signatures resulting from the environmental exposure of the viral RNAs in sewage as well as PCR, sequencing library layout-specific and sequencing device-specific errors (De Maio *et al.* 2020, Turakhia *et al.* 2020, Jacot *et al.* 2021).

Existing standard metagenomic simulators do not attempt to capture all the characteristics seen in the amplicon sequencing protocols used for SARS-CoV-2 such as the ARTIC community protocols (Tyson *et al.* 2020, https://artic.network/ncov-2019, https://artic.network/resources/ncov/ncov-amplicon-v3.pdf) widely used to prepare samples for Illumina sequencing platforms. For example, InSilicoSeq (Gourlé *et al.* 2018) is intended to produce shotgun metagenomic sequences; and while Grinder (Angly *et al.* 2012) can simulate amplicon sequencing, it cannot be tuned to produce a bespoke amplicon distribution and does not produce realistic sequencing quality scores. The simulation tool ART (Huang *et al.* 2011) can also generate reads for amplicons, but only in equal proportions. Studies (Baaijens *et al.* 2022, Gafurov *et al.* 2022, Valieris *et al.* 2022, Sapoval *et al.* 2023) have demonstrated the need for a dedicated wastewater SARS-CoV-2 sequencing simulator. Each, however, performed its own simulations for its specific use case, with most simulators limited to simplified scenarios with uniform amplicon abundances and only read errors (e.g. Baaijens *et al.* 2022, Gafurov *et al.* 2022, Kayikcioglu *et al.* 2023), or recreating the read-depth variation of one specific real experiment (e.g. Valieris *et al.* 2022, Sapoval *et al.* 2023). Both cases have drawbacks, either risking overfitting to one dataset or omitting important characteristics of real-life data such as PCR errors and variable amplicon abundances. Table 1 shows a comparison of some of these simulation tools.

**Table 1.** Comparison of metagenomic simulator features.

Program	Language management	Sequencing technology	Illumina layout	Output file type	Mix.	Amps.	VAA	Ampl. bias	SNP & indel errors		Quality scores
									HF	Reads	
SWAMPy	python; docker	Illumina	PE	FASTQ	✓	✓	✓	✓	✓	✓	✓
ART (Huang *et al.* 2011)	C++, Perl; conda	Illumina, 454, SOLiD	PE	FASTQ, SAM	—	✓	—	—	—	✓	✓
InSilicoSeq (Gourlé *et al.* 2018)	python; conda, docker, pip	Illumina	PE	FASTQ	✓	—	—	✓	—	✓	✓
ww_simulations (Kayikcioglu *et al.* 2023)	python; —	Illumina, ONT	PE	FASTQ	✓	✓	—	—	—	✓	✓
VLQ^1^ (Baaijens *et al.* 2022)	python; conda	Illumina	PE	FASTQ	✓	—	—	—	—	✓	✓
pIRS (Hu *et al.* 2012)	C++, Perl; —	Illumina	PE	FASTQ	—	—	—	✓	—	✓	✓
GemSIM (McElroy *et al.* 2012)	python; —	Illumina, 454	SE, PE	FASTQ, SAM	✓	—	—	—	—	✓	✓
Grinder (Angly *et al.* 2012)	Perl; apt, CPAN	Illumina, 454, Sanger	PE	FASTA, FASTQ	✓	✓	✓	—	—	✓	—^2^
NeSSM (B *et al.* 2013)	C, Perl; —	Illumina, 454	SE, PE	FASTQ	✓	—	—	✓	—	✓	✓
BEAR (Johnson *et al.* 2014)	Perl, python; —	Illumina, 454, Ion Torrent	SE, PE	FASTQ	✓	—	—	—	—	✓	✓
FASTQSim (Shcherbina 2014)	python; —	Illumina, PacBio, IonTorrent, 454	SE	FASTQ	✓	—	—	—	—	✓	✓

Software management systems cited are apt (The Debian Project 2023) https://www.debian.org/doc/manuals/debian-reference/ch02.en.html, conda (Anaconda, Inc. 2023) https://docs.conda.io/en/latest/, CPAN (Hietaniemi 2023) https://www.cpan.org/, docker (Docker Inc. 2023) https://www.docker.com and pip (pip developers 2023) https://pip.pypa.io/en/stable/. Programs with no management system listed are nevertheless open source and available for download and manual installation and use. Sequencing technologies cited are Illumina, Ion Torrent, Oxford Nanopore Technologies, Pacific Biosciences, Roche 454 (all described by Slatko *et al.* (2018)), and Sanger sequencing and ABI SOLiD (Kircher and Kelso 2010). Illumina layout indicates single ended (SE) or paired end (PE) reads. Output file types are described by (Zhang 2016). Mix. indicates whether the program accepts a list of genomes to simulate a mixed sample. Amps.: can the program simulate amplicon sequencing protocols. VAA: are variable (i.e. non-uniform) amplicon abundances modeled. Ampl. bias: whether differential amplification success is modeled, based on (e.g.) reads’ GC content. Regarding SNP and indel errors, HF denotes high-frequency errors, i.e. recurring position-dependent errors, possibly appearing at the same position across different amplicons, as described in Section 2; whereas Reads denotes independent per-read errors, e.g. from sequencing device error.

1 VLQ is a tool for analyzing wastewater sample sequence data for SARS-CoV-2 variant quantification; it has an unnamed simulation tool available as part of its benchmarking code.

2 Output from Grinder can be FASTQ-formatted, but the quality scores serve only to indicate whether or not each position incurred a sequencing error.

Our simulator, SWAMPy (**S**imulating SARS-CoV-2 **W**astewater **A**mplicon **M**etagenomes with **Py**thon), is intended to produce a realistic set of reads that might be generated through multiplex PCR of a wastewater sample, and sequenced by an Illumina sequencer. We model the following scenario:

1. Different viral genomes coming from a human population contaminate wastewater systems, creating a mixture of virus variants which is then captured in a wastewater sample. At this stage, viral RNAs are exposed to RNA degradation and there is a variation in variant abundance in the mixture.
2. After sample collection, PCR amplification of whole viral genomes in segments using a pre-defined primer set results in an amplicon population. At this stage, amplicons gain PCR errors and there is further variation in amplicon abundance due to differential amplification success of the primers of a given primer set.
3. These amplicons are then sequenced on an Illumina device, creating paired-end reads of a fixed length. At this stage, sequencing errors appear.

## 2 Materials and methods

**Figure 1.**
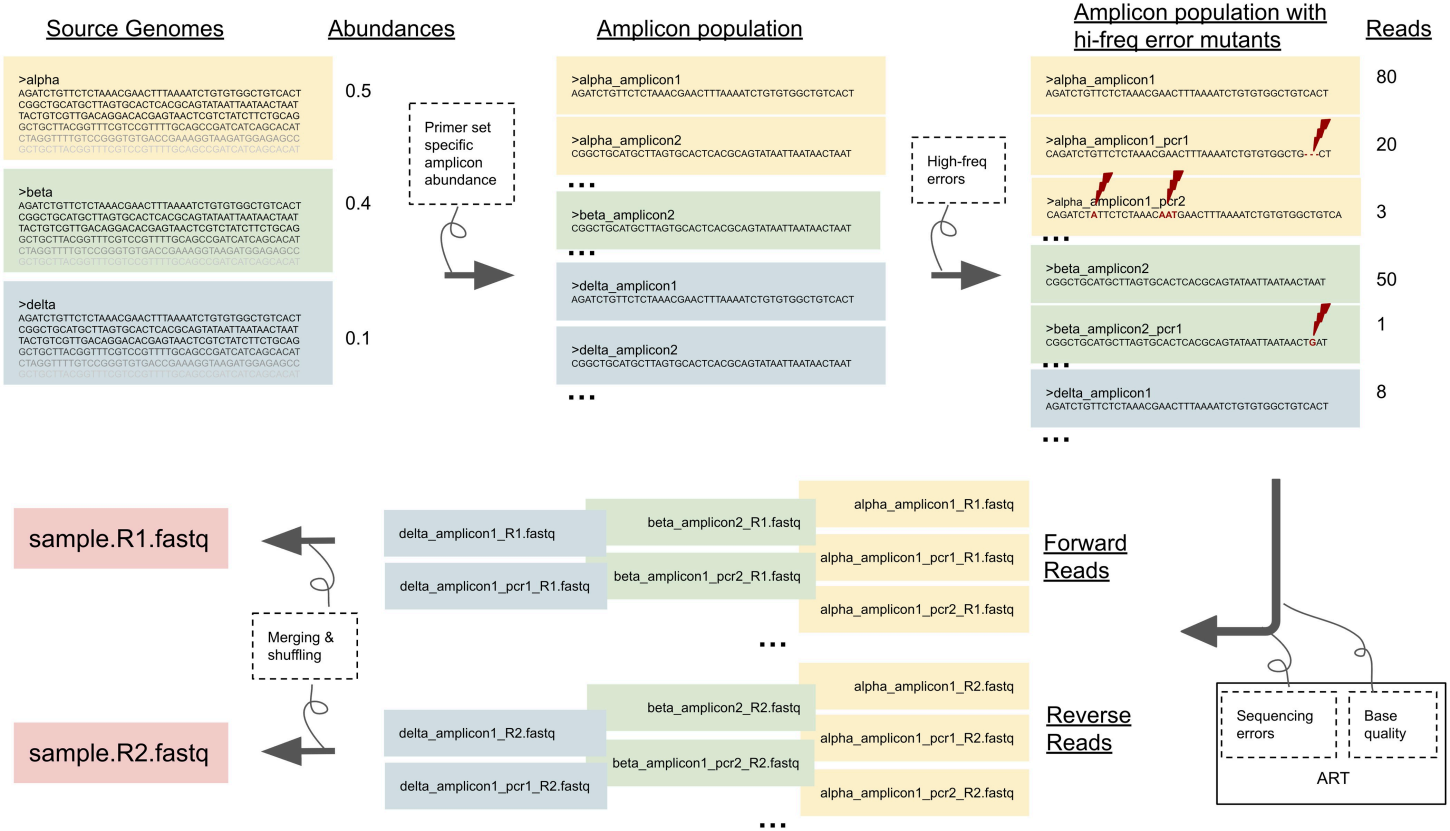
Summary of the SWAMPy workflow. Clockwise from top left, SWAMPy takes as input the genomes of the variants to be represented in the simulated wastewater sample, as well as information on the relative abundances of the variants in the simulated mixture. Source or input genomes are sliced according to a primer set to create a reference amplicon population, and amplicon read depths are adjusted to fit the amplicon abundance distribution of the given primer set (see Supplementary Material) while taking into account a user-defined parameter which reflects the quality of the samples. The amplicon population is then further diversified by the addition of PCR mutants bearing different kinds of high-frequency errors, using parameters estimated from real data (see Supplementary Material). The resulting reference and mutant amplicons, with corresponding read counts, are passed to the ART_illumina program of ART (Huang *et al.* 2011) to model the Illumina sequencing step, where sequencing errors and base qualities are simulated. Finally, reads are merged and shuffled to create mixed-variant forward and reverse FASTQ files.

The overall workflow of SWAMPy can be seen in Fig. 1. The four basic steps of our software pipeline are detailed as follows:

1. Create an initial amplicon population.
2. Simulate the number of DNA fragments (copies) per amplicon.
3. Simulate high-frequency errors by mutating amplicons in the amplicon population. “High-frequency errors” is an umbrella term we use for all substantially recurrent mismatches between the nucleotide sequences of input viral lineages and output reads, which might be caused by a number of phenomena such as RNA degradation, PCR errors, or alternative binding of amplicon primers (De Maio *et al.* 2020). Standard sequencing errors, simulated in the next step, are not considered high-frequency errors because they are randomly distributed across the genome, while high-frequency errors are position-specific (see Methods Section 1.3) and as such might have a higher potential of causing lineage inference errors.
4. Simulate sequencing reads using ART.

### 2.1 Create an initial amplicon population

The software assumes that the user has supplied a set of input SARS-CoV-2 genomes, which we refer to as source genomes, and has selected a supported primer set among ARTIC V1, V4, and V5 (Tyson *et al.* 2020), or Nimagen V2 (Coolen *et al.* 2021) https://pubmed.ncbi.nlm.nih.gov/34619382/. Alternatively, users can supply custom primer panels. On the basis of this selection, amplicons are extracted from each genome as follows. First, we use Bowtie 2 (Langmead and Salzberg 2012) to align the primers (forward and reverse complement) to each virus genome to detect primer binding positions on the source genomes. In a very crude approach to account for amplicon dropouts, if a primer does not align well with a viral genome (i.e. Bowtie 2 does not find a match exceeding its—score-min threshold), the corresponding amplicon is not produced. Next, we slice the source genomes from the primer binding positions to obtain individual amplicons of each source genome, including the primer sequences.

### 2.2 Simulate numbers of copies per amplicon

To simulate the numbers of copies per amplicon and genome, we offer two versions of a combined Multinomial and Dirichlet model. For either of these models, the user must supply three parameters: total target number of reads *N*, a vector of genome abundances *p_g_* indexed over genomes *g*, and a Dirichlet parameter *c*. The choice of *c* roughly equates to a measure of sample quality: a higher value of *c* (e.g. 200) corresponds to high-quality samples (roughly uniform abundances of amplicons between simulation runs) and a lower value (e.g. 10) to low-quality samples (highly variable amplicon abundances between simulations, with higher rates of amplicon dropout). For the supported primer sets SWAMPy provides an experimentally derived prior on the amplicon proportions *π_a_* indexed over amplicons *a*. As explained in our software documentation, the user has the option of overriding these default priors and supplying their own.

**Model 1** (equal expected amplicon proportions across genomes):

1. Sample genome read counts *N_g_* from $\text{Multinomial}(N,p_{g})$
2. Sample amplicon proportions *p_a_* from $\text{Dirichlet}(c\times \pi_{a})$ to be shared across all genomes
3. For each genome, sample numbers of reads per amplicon per genome as $x_{a,g}∼\text{Multinomial}(N_{g},p_{a})$

**Model 2** (different amplicon proportions across genomes):

1. Sample genome read counts *N_g_* from $\text{Multinomial}(N,p_{g})$
2. For each genome, independently sample amplicon proportions $p_{a,g}$ from $\text{Dirichlet}(c\times \pi_{a})$
3. For each genome, sample numbers of reads per amplicon per genome as $x_{a,g}∼\text{Multinomial}(N_{g},p_{a,g})$

One subtlety in this process is that the numbers of reads do not account for amplicons dropped in the alignment step, which leads to some missing reads. For example, if the model assigns 100 reads to amplicon 1 in genome A, yet a mutation at the primer site of this amplicon causes it to drop out, then the total number of reads produced would be 100 fewer than expected. Hence the actual total number of reads may be less than the target, *N*.

### 2.3 Add high-frequency errors

While we model uniformly distributed sequencing error with the standard bioinformatics tool ART (Huang *et al.* 2011), we also developed a model to generate recurrent mismatches between input viral genomes and output reads, mimicking the effects of a number of phenomena (De Maio *et al.* 2020) such as RNA degradation, PCR mutations, reverse transcription errors, library preparation artifacts, alternative primer binding, etc, which we collectively refer to as “high-frequency errors.” We define high-frequency errors as non-naturally occurring insertions, deletions, and substitutions that non-independently affect multiple reads. Rather than trying to distinguish different independent sources of recurrent errors, we use a small number of distributions to model the combined effect of all these phenomena. We classify high-frequency errors as unique or recurrent with respect to their presence across different source genomes in the mixture. Recurrent errors are the ones that are present in all source genomes in the simulated mixture, consistent with our observation of individual errors affecting multiple real wastewater sequencing experiments. These might originate, e.g. from genomic positions particularly susceptible to degradation, or context-dependent PCR errors (Meyerhans *et al.* 1990, Potapov and Ong 2017). In contrast, unique high-frequency errors are present in only one of the genomes in the mixture, e.g. due to context-dependent PCR errors, or low-rate RNA degradation.

### 2.4 Sampling high-frequency errors

To simulate high-frequency errors in SWAMPy, we first create a table like that shown in Table 2 containing all the sampled high-frequency errors to be introduced.

**Table 2.** Example simulated high-frequency errors.

Type	rec/u	Genome	len	pos	ref	alt	VAF	amp
subs	rec	g1, g2, g3	1	20 000	A	T	0.1	70, 71
subs	u	g2	1	530	T	G	0.2	3
ins	rec	g1, g2, g3	7	245	A	AGCG	0.9	2
del	u	g3	3	230	AGCT	A	0.6	2

Abbreviations: rec, recurrent; u, unique; subs, substitution; del, deletion; ins, insertion; amp, amplicon number; len, length; alt, alternative allele; pos, genomic position; g*N*, SARS-CoV-2 variant genome.

1. The number of each type of error to be introduced is sampled from Poisson(L×R) where *L* = 29 903 is the length of the Wuhan reference genome Wuhan-Hu-1 (Wu *et al.* 2020) and *R* is the error rate of the given type of error (insertion, deletion, or substitution, each either unique or recurrent). This Poisson distribution approximates the Binomial(L,R) distribution since error rates are typically low. Error rates are user-definable for each of the six types of error, with default values estimated from real wastewater experiments (see Supplementary Material).
2. A genomic position for each error is sampled randomly without replacement from Wuhan-Hu-1. For unique errors, one of the source genomes is randomly assigned with sampling weights equal to the genome abundances in the mixture. Moreover, if more than one amplicon spans the previously determined error position, a unique error is assigned to only one of them. Recurrent errors are assigned to all source genomes and overlapping amplicons.
3. An error length is assigned to each error. The error length is always 1 for substitutions, while for deletions it is sampled from a geometric distribution with parameter *n*; higher *n* will result in shorter deletions. For insertions, the error length is sampled from Uniform(m) where *m* is the maximum insertion length. Error length parameters *n* and *m* can be defined by the user, with their default values obtained from real data (see Supplementary Material).
4. An alternative allele is created for each error. For substitutions, it is a random single nucleotide that is different from the reference genome, and for insertions, it is a sequence of randomly sampled nucleotides of the previously determined error length.
5. A variant allele frequency (VAF), *f*, is sampled for each error from a Beta(α,β) distribution. The Beta distribution parameters are similarly user-definable, separately for unique and recurrent substitutions, insertions, and deletions. Assigned VAF values are the expected VAF of the recurrent errors in the final mixture, while for unique errors, the expected value of the VAF in the final mixture will be the product of the assigned VAF *f* and the corresponding amplicon abundance *π_a_*.

### 2.5 Apply sampled errors to simulated amplicons

After we compile the table that contains all simulated errors, we process each source genome *g* and each amplicon *a* in the amplicon population that we previously created. For each *a*, *g*:

1. Errors that affect genome *g* and amplicon *a* are selected from the error table.
2. Because simulated error positions are based on the Wuhan-Hu-1 reference and a variant amplicon in a wastewater sample may contain indels, the amplicon sequences are aligned to Wuhan-Hu-1 using Bowtie 2 (Langmead and Salzberg 2012), and the positions of the errors within the amplicon are determined.
3. For each error *e*, the number of reads in which *e* is present is determined by sampling a read count *n_e_* from $\text{Binomial}(x_{a,g},f_{e})$, where $x_{a,g}$ is the total read count of amplicon *a* for genome *g* as described in Section 1.2, and *f_e_* is the VAF of the error as determined in Section 1.3.1.
4. For each possible combination *i* of high-frequency errors affecting genome *g* and amplicon *a*, a read count *n_i_* is randomly sampled respecting individual read counts of the errors. We make no attempt to simulate correlations among the errors on amplicons as simulating error inheritance for each amplicon is computationally too expensive and we assume errors on an amplicon are independent.
5. Finally, for each combination *i* of errors affecting *a* and *g*, a new corresponding modified amplicon sequence is created.

### 2.6 Simulate read sequencing using ART

To create a set of simulated paired-end Illumina reads from each amplicon, each with a given read count, we use the program ART (Huang *et al.* 2011). We use ART’s paired-end amplicon mode, as well as the noALN and maskN flags. These settings create paired-end reads of customizable length (default 250 bp) and faithfully transcribe any “*N*” characters appearing within the amplicons. We use a set of default error rates and quality score profiles tuned for the Illumina MiSeq V3 sequencer, though the ART package has options available for other platforms and read lengths. A full list of the flags used is in the Supplementary Material.

Finally, we use a custom script based on ubiquitous bash utilities to concatenate all of the FASTQ read files, and shuffle their order to avoid potential biases in case any downstream application software can be influenced by read ordering.

## 3 Results

The source code of our python implementation of SWAMPy, together with the program documentation and exemplar files is available under the GPL-v3 license at https://github.com/goldman-gp-ebi/SWAMPy.

### 3.1 SWAMPy implementation

SWAMPy takes as input a multi-FASTA file containing the SARS-CoV-2 variant genomes that will be present in the simulated wastewater sample, as well as a file that contains the relative abundances of these variants in the mixture. For ease of use, other input files (primer-set-specific sequence files, and primer-set-specific amplicon distribution files) were wrapped with a single—primer-set parameter which loads the corresponding input files for the specified primer set. As of June 2024, there are four supported primer sets: ARTIC V1, V4, and V5 (Tyson *et al.* 2020), and Nimagen V2 (Coolen *et al.* 2021). There are many command line parameters that allow fine control of the program such as the amplicon pseudo counts parameter *c* that reflects the quality of the wastewater sample as described in Section 1.2, the target number of simulated reads, and error rates, VAF and lengths of high-frequency errors as described in Section 1.3.1. The full list of command line interface arguments and their explanations are available on the GitHub wiki page: https://github.com/goldman-gp-ebi/SWAMPy/wiki/CLI-arguments.

An example SWAMPy run takes 300 seconds to complete and reaches 700 MB of max memory when run with default parameters (three SARS-CoV-2 variants and default error rates and 100 000 total read counts) on a single thread of an Intel Xeon Gold 6336Y 2.40 GHz CPU.

SWAMPy produces five output files by default:

- FASTQ files of the simulated forward and reverse reads, matching Illumina standards
- A table that shows the abundance of each wild-type amplicon after the randomness in amplicon copy number sampling (as described in Section 1.2) was applied
- A VCF file that contains all the intended high-frequency errors from the error table described in Section 1.3.1
- A log file

**Figure 2.**
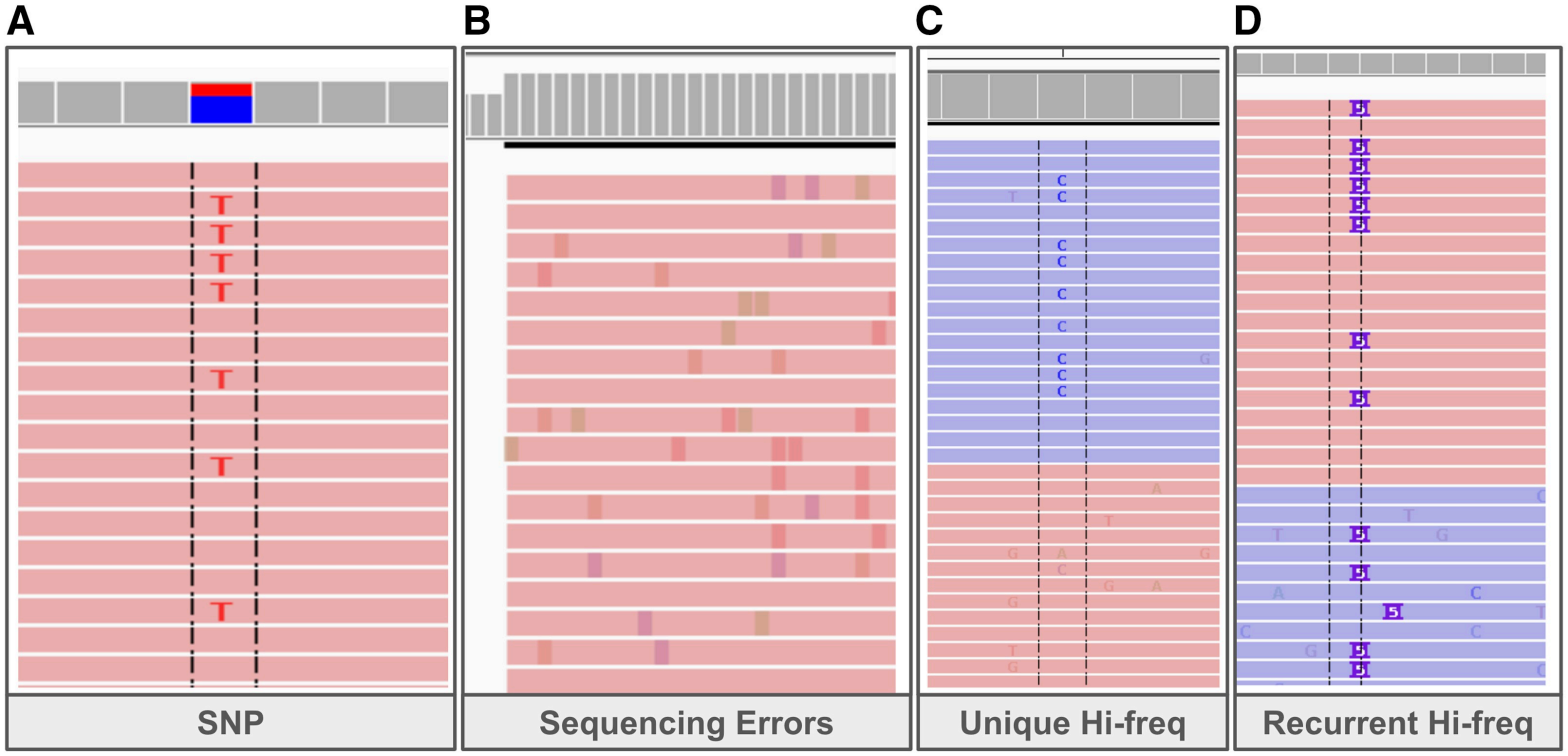
IGV (Robinson *et al.* 2011) images of SWAMPy simulated reads. Reads come from time point 53 of the simulations described in Section 3.3, involving SARS-CoV-2 Alpha, Delta, and Omicron variants. Uppermost portion is the coverage track of IGV. Below that, horizontal bars show forward and reverse reads in “link supplementary alignments” mode of IGV. Black dashed lines are IGV’s optional “center line,” aiding visual perception of the alignment of bases; faded colors indicate low-quality bases. (**A**) C → T SNP at position 7124 in the Delta variant, is not present in the Alpha and Omicron variants. (**B**) Sequencing errors added by ART. This image (zoomed out, with variant bases indicated by color) is from a read end, where sequencing error density is often higher with Illumina sequencing. (**C**) Unique high-frequency error. This only appears in one read direction and, despite this exemplar being chosen to be in an amplicon overlap region (not shown), only one of the amplicons carries the error. (**D**) Recurrent high-frequency error (insertion of length 5) appearing in both read directions and in both amplicons covering the chosen region (not shown).

Alignment images of simulation outputs illustrate that the major characteristics of wastewater data are present in the simulated data (Fig. 2) such as overlapping amplicons, different kinds of errors, and read depth variation across the genome.

### 3.2 Benchmark with real data

**Figure 3.**
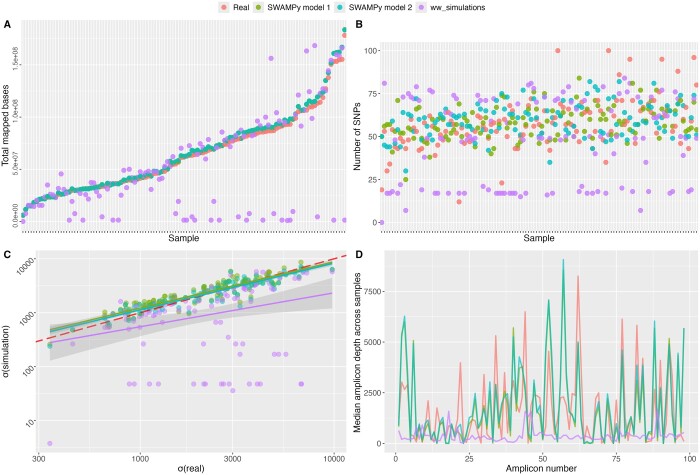
Comparison of real and simulated wastewater sequencing data. We simulated wastewater sequencing data using SWAMPy and ww_simulations, mimicking a real dataset, and then compared features of real and simulated datasets. (**A**) Total simulated bases mapped to the Wuhan-Hu-1 reference genome (*Y*-axis), against real ones (*X*-axis), per sample. The drop in coverage in some simulated ww_simulations samples appears to be due to strict primer binding requirements. (**B**) Numbers of high-frequency SNPs (VAF >20% and depth of alternative allele > 30) per sample. Samples are ordered by total depth in the real data. (**C**) Standard deviations of depth across the genome, on a ${\;\text{log}\;}_{10}$ scale, of simulated data (*Y*-axis) against real data (*X*-axis). Each dot represents one sample. The red dashed line indicates equality between the simulated and observed data, thick lines are lines of best fit. (**D**) Median coverage across samples (*Y*-axis) for each amplicon (*X*-axis). The two SWAMPy models are not distinguishable due to strong overlap. Unlike SWAMPy, the ww_simulations package assumes uniform coverage prior across amplicons.

We benchmarked SWAMPy models 1 and 2, as well as the amplicon wastewater simulation tool ww_simulations (Kayikcioglu *et al.* 2023) https://github.com/CFSAN-Biostatistics/ww\_simulations/ using 120 real SARS-CoV-2 wastewater samples listed in the Supplementary Material. These samples formed part of a Houston SARS-CoV-2 surveillance project and were sequenced using the ARTIC V3 protocol, which differs from V1 by the introduction of 11 pairs of alternative primers. We used the SARS-CoV-2 deconvolution tool Freyja (Karthikeyan *et al.* 2022) to estimate the proportions of the dominant lineages from each real sample (those above 2% abundance). We then generated synthetic mixtures of these same lineages and proportions with each simulation tool considered—SWAMPy models 1 and 2, and ww_simulations. Real and simulated read sets were then mapped back to the Wuhan-Hu-1 reference genome to calculate variant allele frequencies and amplicon proportions. SWAMPy more faithfully captured the variation in amplicon proportions than ww_simulations (Fig. 3D, Supplementary Fig. 3), and overall read depths across the genome (Fig. 3A and C). Variant allele frequencies generated by SWAMPy were also more similar to the real dataset than ww_simulations (Fig. 3B, Supplementary Fig. 4). For all simulators, we tuned three parameters, selecting a read length of 150 bp, and fragment size and standard deviation of 155 and 75, respectively. Other parameters could have been tuned to generate amplicon distribution mean and variance customized for this wastewater dataset. Although SWAMPy models 1 and 2 both performed similarly when comparing bulk properties of the VAF and amplicon abundance distributions, the difference can be seen when looking at amplicon distributions at a lineage-resolved level (Supplementary Fig. 5). For this dataset, both SWAMPy and ww_simulations seem to generate reads with overly pessimistic error rates. Both tools rely on the short read simulator ART_illumina for this task. When we turned off read errors in ART within SWAMPy, the number of simulated low-frequency variants became much closer to those observed in real data (Supplementary Fig. 4). This suggests that, in addition to SWAMPy’s parameters, realistic simulations might often need the tuning of ART’s parameters as well.

### 3.3 Use case

**Figure 4.**
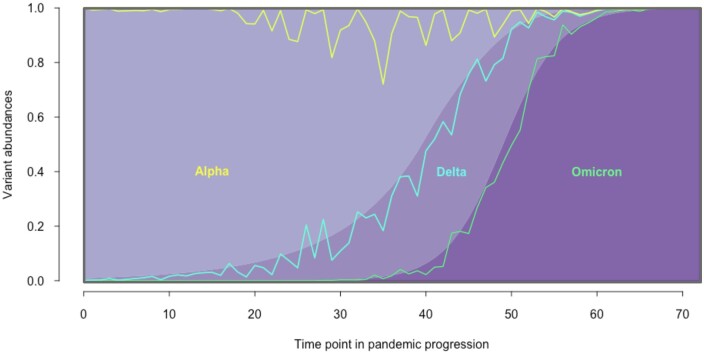
Progression of a simulated pandemic. Sequencing of wastewater samples at 73 time points was simulated with SWAMPy, and corresponding Freyja estimations of SARS-CoV-2 variant abundances were made. Background colors represent the simulated values and lines represent the Freyja estimations. Lines generally follow the boundaries between the shaded areas, suggesting broadly accurate variant proportion estimates from Freyja. The region above the topmost jagged line (yellow) shows the sum of non-simulated variants (i.e. false positive variant detection) that Freyja erroneously inferred.

We used SWAMPy to simulate 73 time points throughout the course of a hypothetical SARS-CoV-2 pandemic where the Alpha (B.1.1.7) variant starts out dominant before Delta (AY.4) rises in frequency and then Omicron (BA.1.1) emerges and takes over (Fig. 4; see the Supplementary Material for the SWAMPy options used; for the exact abundances at each time point, see Supplementary Material). Then we used a downstream application program, Freyja, which is designed to detect SARS-CoV-2 variants and their relative abundances from sequencing data obtained from wastewater samples (Karthikeyan *et al.* 2022). We observe that Freyja is quite successful in demixing the simulated data overall in this relatively complex scenario, though it sometimes inferred the presence of variants that are not specified in the simulated mixture, occasionally with high frequencies.

## 4 Conclusions

We have shown that SWAMPy is a viable simulation tool for SARS-CoV-2 wastewater metagenomes, building on the simulator ART but much better suited to the modeling challenges idiosyncratic to SARS-CoV-2 metagenomes such as high-frequency errors and irregular amplicon abundance profiles. Both of these models are based on real abundance and error data, from a large number of *in vitro* whole-genome amplicon sequencing experiments, detailed in the Supplementary Material. Compared with other metagenomic simulators, many of which support a range of complex features such as chimeric amplicons and shotgun metagenomic reads, SWAMPy aims to fill a niche created by metagenomic amplicon studies such as wastewater surveillance of SARS-CoV-2. This niche seems important given the disparity between features available in most general-purpose metagenomic simulators (Table 1) and the requirements of tools being developed for wastewater studies.

Our simulator supports three versions of the ARTIC protocol, which at present is the most prevalent sequencing protocol for SARS-CoV-2 metagenomes, and the Nimagen V2 protocol. We will strive to support future iterations of these, as well as new superseding protocols as they arise in the future. There are other areas where we hope to make improvements to modeling and usability, such as supporting a greater range of sequencing platforms and more closely matching amplicon dropout rates with experimental findings. It might also be beneficial in the future to account for additional complexity in high-frequency error models, such as specific sources of errors (e.g. PCR amplification and RNA degradation), and empirically derived position-specific error rates.

We hope that our simulation tool will prove valuable by providing non-trivial test cases, especially for strain-resolving SARS-CoV-2 metagenomics algorithms, and for creating control case data for researchers working on SARS-CoV-2 wastewater studies. Wastewater surveillance can provide a cheaper alternative to widespread sequencing of clinical SARS-CoV-2 samples, and it is our hope that through appropriate modeling and simulation of the processes involved in amplicon sequencing of wastewater, these data can be leveraged to their full potential in aiding public health.

## Supplementary Material

btae532_Supplementary_Data

## Data Availability

Nimagen and synthetic ARTIC amplicon sequences are available at the European Nucleotide Archive, BioProject accession PRJEB53222. Real wastewater samples used for parameter estimation, sequenced using the ARTIC v3 protocol are available on request and with permission from the Joint Biosecurity Centre and University of Liverpool. Summarised data (e.g. amplicon counts) are available through GitHub at https://github.com/goldman-gp-ebi/SWAMPy/tree/main/supplementary_files. The SWAMPy parameters used to generate the 73 simulated time points are available in the supplementary information. The samples used for validation and comparison with ww_simulations are available at ENA BioProject accession PRJNA796340, with the exact samples used listed in the supplementary information file. We added data availability information on our GitHub page: https://github.com/goldman-gp-ebi/SWAMPy/blob/main/manuscript_data_availability.md.
