# Supplementary material for: SWAMPy: simulating SARS-CoV-2 wastewater amplicon metagenomes
Source: Bioinformatics. 2024 Sep 3;40(9):btae532. doi: 10.1093/bioinformatics/btae532 (PMC11401744; doi:10.1093/bioinformatics/btae532)
Supplement: btae532_Supplementary_Data [file btae532_supplementary_data.pdf]

Supplementary Note

SWAMPy: Simulating SARS-CoV-2 Wastewater Amplicon Metagenomes

William Boulton,[1,2,†] **Fatma Rabia Fidan**,[1,3,4,†] **Hubert Denise**,[5,‡] **Nicola De Maio**[1] **and Nick Goldman**[1,*]

[1]European Molecular Biology Laboratory, European Bioinformatics Institute (EMBL-EBI), Hinxton, Cambs., CB10 1SD, U.K., [2]Department of Computing Sciences, University of East Anglia, Norwich Research Park, Norwich, Norfolk, NR4 7TJ, U.K., [3]Department of Biological Sciences, Middle East Technical University, 06800 Ankara, Turkey, [4]Cancer Dynamics Laboratory, Francis Crick Institute, 1 Midland Road, London, NW1 1AT, U.K. and [5]UK Health Security Agency, Department of Health and Social Care, Nobel House, London, SW1P 3HX, U.K.

[†]These authors contributed equally.

[‡]Current address: Sensica Ltd., RILD Building, Barrack Road, Exeter EX2 5DW, U.K.

[*]Corresponding author. goldman@ebi.ac.uk

Parameter Estimation and Simulation Experiment Parameters

Amplicon abundance estimation

To estimate Dirichlet parameters for amplicon abundances of the ARTIC v3 and Nimagen v2 primer schemes, we summed amplicon counts over a number of experiments, using results from both synthetic SARS-CoV-2 sequences and real wastewater data. In either case, viral RNA was reverse transcribed before being amplified using each of these two primer schemes. The spreadsheets at `https://github.com/goldman-gp-ebi/SWAMPy/tree/main/supplementary_files` provides a summary of amplicon counts through a number of experiments. We excluded amplicon counts in experiments where there was an obvious systematic bias: in some experiments one of the two primer pools failed, and we discarded these results. Other amplicons failed to amplify in the synthetic virus genomes; this was due to the synthetic genomes being produced in 5 kbp chunks. We again discarded these amplicon counts. Finally we normalised our counts' values so that they summed to 1.

For the Artic V4 primer scheme, we used an amplicon abundance profile based on a single experiment (Joshua Quick, personal communication). The coverage file for this experiment is again provided at `https://github.com/goldman-gp-ebi/SWAMPy/tree/main/supplementary_files`.

ART Parameters

To simulate sequencing errors we used the program **ART_illumina** (part of the ART suite of simulation tools (Huang et al., 2011)), with the following command-line parameters:

```
-amplicon
-paired
-noALN
-maskN 0
-seqSys SEQ_SYS
-len READ_LENGTH
-rcount NUMBER_OF_READS
-rndSeed SEED
-in INPUT_AMPLICON_FASTA
-out OUTPUT_FASTQ_FILENAME
-qShift 0 -qShift2 0
```

SEQ_SYS, READ_LENGTH, and NUMBER_OF_READS are set by the user. SEED, INPUT_AMPLICON_FASTA, and OUTPUT_FASTQ_FILENAME are changed for each amplicon of each genome that is being simulated.

When simulating amplicon fragmentation, we remove the parameter `-amplicon` and instead use `-mflen` and `-sdev` to specify mean fragment length and standard deviation. Quality scores can also be adjusted through the `-qShift` parameter.

To define the default high-frequency error rates in SWAMPy, we performed an error characterisation analysis on 121 real wastewater sequencing datasets. Wastewater sequencing experiments were conducted by members of JBC-led Wastewater Genomics collaboration. 12 of the 121 samples are mixtures of synthetically produced SARS-CoV-2 variant genomes, which mimic wastewater samples. Their ENA accessions are:
ERR10084556, ERR10084565, ERR10084558, ERR10084543, ERR10084590, ERR10084581, ERR10084585, ERR10084577, ERR10084594, ERR10084592, ERR10084549, ERR10084547, ERR10084599, ERR10084595, ERR10084586, ERR10084579, ERR10084589, ERR10084564, ERR10084588, ERR10084562. 109 of them were sampled from wastewater in the UK.

© The Author . Published by Oxford University Press. All rights reserved. For permissions, please e-mail: journals.permissions@oup.com

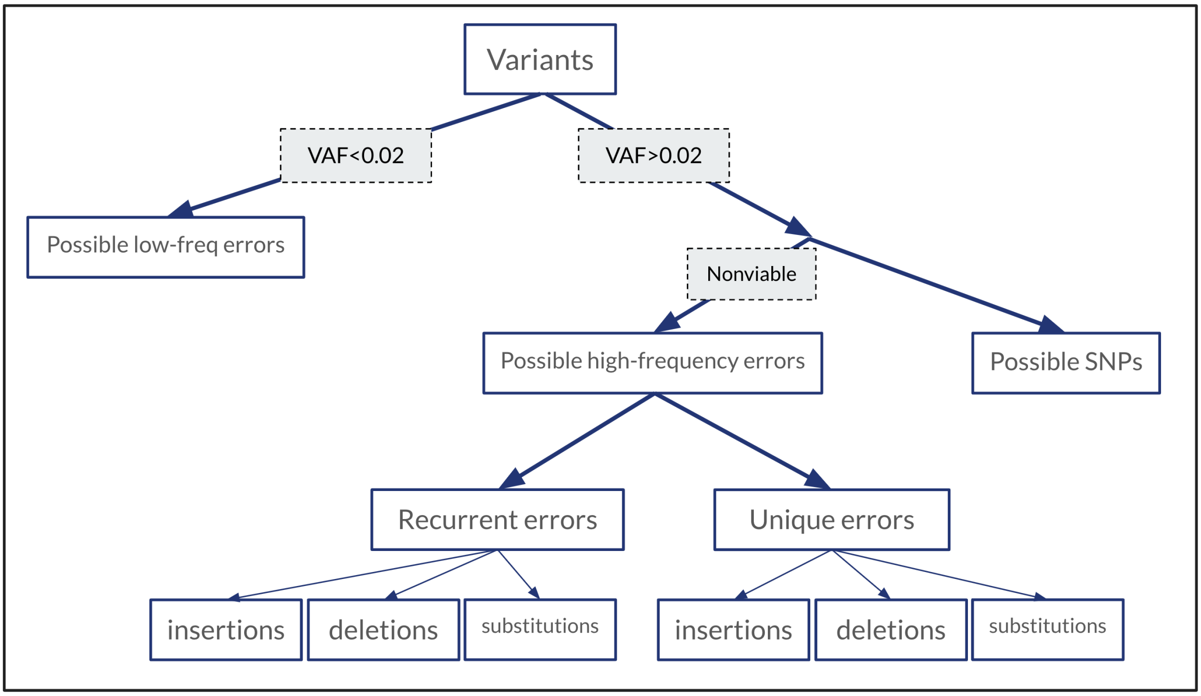

Fig. 1: **Variant classification criteria.**

For each sample of these datasets, we mapped raw reads to the Wuhan-Hu-1 (Wu et al., 2020) reference genome using Bowtie 2.4.4 (Langmead and Salzberg, 2012). We then used bcftools mpileup 1.13 (Danecek et al., 2021) to obtain VCF files. We did not perform a separate variant calling as we are interested in errors as opposed to SNPs and needed every discrepancy between the reference genome and our sample reads; henceforth will refer to such differences as "variants". We filtered out positions with a read depth (DP) < 10 and with < 5 reads supporting the alternative allele (AD). The remaining variants are classified into different categories as summarised in Fig. 1. First, if the variant allele frequency (VAF) of a variant is low, in particular lower than 0.02, we filtered out that variant since such low-frequency variants might be the result of standard sequencing errors (Stoler and Nekrutenko, 2021) and are unlikely to affect downstream analyses. The remaining variants included real polymorphisms between different SARS-CoV-2 variants, as well as possible high-frequency errors, which is the group of errors of interest. To identify likely high-frequency errors, we focused on putative nonviable mutations, since nonviable mutations cannot be real polymorphisms.

High-frequency errors parameter estimation

We define putative nonviable mutations as nonsense (stop codon) substitutions or indels of length not a multiple of three found on ORF1ab and S open reading frames. We excluded other smaller and less characterized genes from the analysis since these can present viable nonsense mutations (Delbue et al., 2021; Jungreis et al., 2021). To further eliminate possible real polymorphisms, we also excluded a portion from the $3'$ ends of the ORF1ab and S open reading frames since nonsense mutations could be tolerable there. The exact positions included are 266–12000, 13465–20000 and 21563–25000. We further divided the high-frequency errors as either recurrent or unique based on if they appeared in more than one wastewater sample. We subdivided both recurrent and unique errors into insertions, deletions and substitutions subclasses, and estimated default rates of these events by counting the observed variants in each class and normalizing by the number of genome positions with sufficient coverage and correcting for the expected proportions of high-frequency errors that would not have been identified (indels with length multiple of three and non-stop codon substitutions). A more exhaustive description of the approach to infer high-frequency error rates and lengths is given in the following subsections.

Indel error length distributions

Following the length distributions observed in our putative high-frequency indel errors (see figure 2), we model high-frequency indel error lengths as:

- Insertions: uniform distribution with minimum length 1 and maximum 14 (the minimum and maximum lengths found in real data) $U(1, 14)$.
- Deletions: geometric distribution with parameter p, Geometric(p).

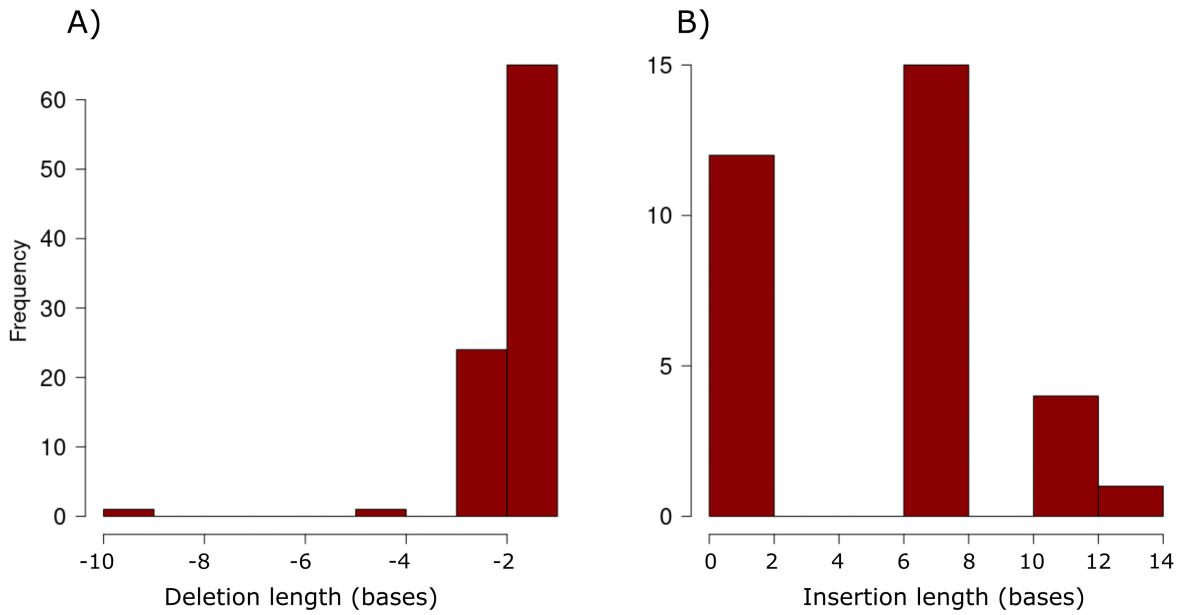

Fig. 2: **Histograms of putative indel error lengths observed in real data.** A) Deletions; B) Insertions.

We could not use a standard distribution fitting technique to estimate the indel length geometric distribution parameter p because we cannot observe putative high-frequency error deletions with length multiple of three. Instead, to avoid potential biases due to the missing data, we estimate p using only the counts of putative high-frequency deletion errors of length one and two. Since under Geometric(p) the ratio of the probability of lengths $l = 2$ and $l = 1$ is

$$\frac{P(l=2)}{P(l=1)} = \frac{(1-p)p}{p} = 1-p \tag{1}$$

We use the estimator

$$\hat{p} = 1 - \frac{D_2}{D_1} \tag{2}$$

where D_1 and D_2 are the observed counts of putative high-frequency deletion errors of length 1 and 2 respectively.

High-frequency error rate estimation

We estimated six separate high-frequency error rates, one for each of the six classes of high-frequency errors. For each high-frequency error class i, we call C_i its putative error count observed in the real data, and we estimate its rate r_i as

$$\hat{r_i} = f_i \frac{C_i}{L} \tag{3}$$

where f_i is the missing data correction factor for class i, and L is the total number of genome positions, across all real datasets, that we considered when looking for putative high-frequency errors. For substitutions, f_i corrects for the fact that only nonsense variants could be identified as putative high-frequency errors, while high-frequency errors causing other types of substitutions were not counted as they could not be distinguished from real polymorphisms. Consequently, we defined the correction factor f_i for substitutions as M/M_n, where M is the number of all possible substitutions across the considered portion of the reference SARS-CoV-2 genome (Wuhan-Hu-1, Wu et al., 2020), so $M = 3L$ where L is the length of the considered portion of reference genome; and M_n is the number of possible nonsense mutations across the same portion of the reference genome.

For insertions, the correction factor f_i is simply 3/2 because we assume a uniform high-frequency insertion length distribution and because we did not count insertions with length multiple of three among putative high-frequency insertion errors.

For the deletion correction factor we took a similar approach, but accounting for the assumed geometric distribution of lengths. In a geometric distribution with parameter p, the total probability of all multiples of three is given by

$$\begin{aligned}\sum_{i=1}^{\infty} p(1-p)^{3i-1} &= p(1-p)^2 \sum_{i=1}^{\infty} (1-p)^{3i-3} \\ &= p(1-p)^2 \sum_{i=1}^{\infty} ((1-p)^3)^{i-1} = \frac{p(1-p)^2}{1-(1-p)^3}\end{aligned} \tag{4}$$

where for the last step we used the identity $\sum_{x \geq 0} x^i = (1-x)^{-1}$. The total probability of the lengths that we do observe is 1 minus this value, i.e. $1 - p(1-p)^2/(1-(1-p)^3)$, and therefore we use as correction factor f_i of high-frequency deletions its inverse:

$$\frac{1}{1 - \dfrac{p(1-p)^2}{1-(1-p)^3}} = \frac{p^2 - 3p + 3}{2-p} \tag{5}$$

High-frequency error frequency distributions

To model the default variant allele frequencies (VAF) of high-frequency errors of each type, we use the Beta distribution (see e.g. Lange, 1995) whose default parameters were estimated from the frequencies of putative high-frequency errors using the method of moments.

Benchmark with real data

As a validation dataset, we used samples from the Houston wastewater project with NCBI BioProject accession PRJNA796340, specifically those using the ARTIC V3 protocol. We filtered these samples, keeping those with a mean sequencing read length of approximately 150. We also removed several samples where ww_simulations failed to run. Below is the list of SRA accessions used: SRR17568720, SRR17568721, SRR17568722, SRR17568723, SRR17568724, SRR17568725, SRR17568726, SRR17568727, SRR17568728, SRR17568729, SRR17568730, SRR17568731, SRR17568732, SRR17568733, SRR17568734, SRR17568735, SRR17568736, SRR17568737, SRR17568738, SRR17568739, SRR17568740, SRR17568741, SRR17568744, SRR17568745, SRR17568746, SRR17568747, SRR17568748, SRR17568749, SRR17568750, SRR17568751, SRR17568752, SRR17568753, SRR17568754, SRR17568755, SRR17568756, SRR17568757, SRR17568758, SRR18328656, SRR18328657, SRR18328658, SRR18328659, SRR18328660, SRR18328661, SRR18328662, SRR18328664, SRR18328666, SRR18328669, SRR18328670, SRR18328671, SRR18328673, SRR18328674, SRR18328675, SRR18328676, SRR18328677, SRR18328679, SRR18328680, SRR18328681, SRR18328682, SRR18328683, SRR18328685, SRR18328686, SRR18328688, SRR18328690, SRR18328691, SRR18328692, SRR18328693, SRR18328694, SRR18328695, SRR18328696, SRR18328697, SRR18328698, SRR18328699, SRR18328700, SRR18328701, SRR18328703, SRR18328704, SRR18328705, SRR18328706, SRR18328707, SRR18328710, SRR18328711, SRR18328712, SRR18328713, SRR18328714, SRR18328715, SRR18328716, SRR18328718, SRR18328719, SRR18328721, SRR18328722, SRR18328723, SRR18328788, SRR18328789, SRR18328790, SRR18328791, SRR18328792, SRR18328793, SRR18328794, SRR18328797, SRR18328799, SRR18328800, SRR18328801, SRR18328802, SRR18328803, SRR18328805, SRR18328806, SRR18328807, SRR18328808, SRR18328809, SRR18328810, SRR18328811, SRR18328812, SRR18328813, SRR18328814, SRR18328815, SRR18328816, SRR18328817, SRR18328818, SRR18328820, SRR18328821.

We ran SWAMPy simulations using the following command-line options:

```
--primer_set a1
--amplicon_distribution dirichlet_1 and dirichlet_2
--nreads NREADS
--read_length 150
--fragment_amplicons
--fragment_len_mean 155
--fragment_len_sd 75
```

and for the read-error-free simulations:

```
--art_qshift 93
```

We ran ww_simulations with the following options:

```
-p artic_v3_nCoV-2019.tsv
-n NREADS
-l 150
```

For each sample, the choice of `NREADS` was determined using the total number of mapped reads in the real data. We downloaded consensus sequences of Pango lineages (Rambaut et al., 2020) from `https://github.com/corneliusroemer/pango-sequences/tree/main` (accessed November 2023), and when running simulations, we used the lineages present in this database that were reported by Freyja (Karthikeyan et al., 2022) above a 2% abundance threshold.

Comparison of SWAMPy Model 1 and Model 2

Models 1 and 2 in SWAMPy differ only in how amplicon proportions are drawn when generating numbers of amplicons for lineages within a single simulation. In model 1, a single distribution of amplicon abundances is generated, and shared across all lineages in the simulation. In model 2, a different amplicon distribution profile is drawn for each lineage.

We showcase sample SRR18328793 to highlight the difference between these two models. Freyja estimated this sample to be a mixture of two significantly different lineages, 43% Alpha (lineage Q.2), 50% Epsilon (B.1.427 and B.1.429) and only 7% other lineages. When simulating SARS-CoV-2 metagenomes using these proportions as input, model 1 produced a highly consistent proportion of Alpha variant reads across amplicons (all close to 43%), especially for amplicons where the total coverage is high. Model 2 produced more erratic proportions of Alpha variant reads across amplicons, even though the total proportion across all amplicons was still 43%. The amount of variability generated is tied to the amplicon pseudocounts parameter; setting this lower than the default value 200 produces yet more variability in the amplicon distributions. The red crosses in panels A and B show the observed proportions of Alpha variant SNPs, compared to Epsilon lineage B.1.427, in the real data: these are the informative loci to distinguish Alpha from Epsilon. In this particular case, model 2 seems to more realistically account for the variation in amplicon abundances across lineages.

Use-case

The exact proportions of the SARS-CoV-2 variants at the 73 time points in our simulations are shown in Table 1.

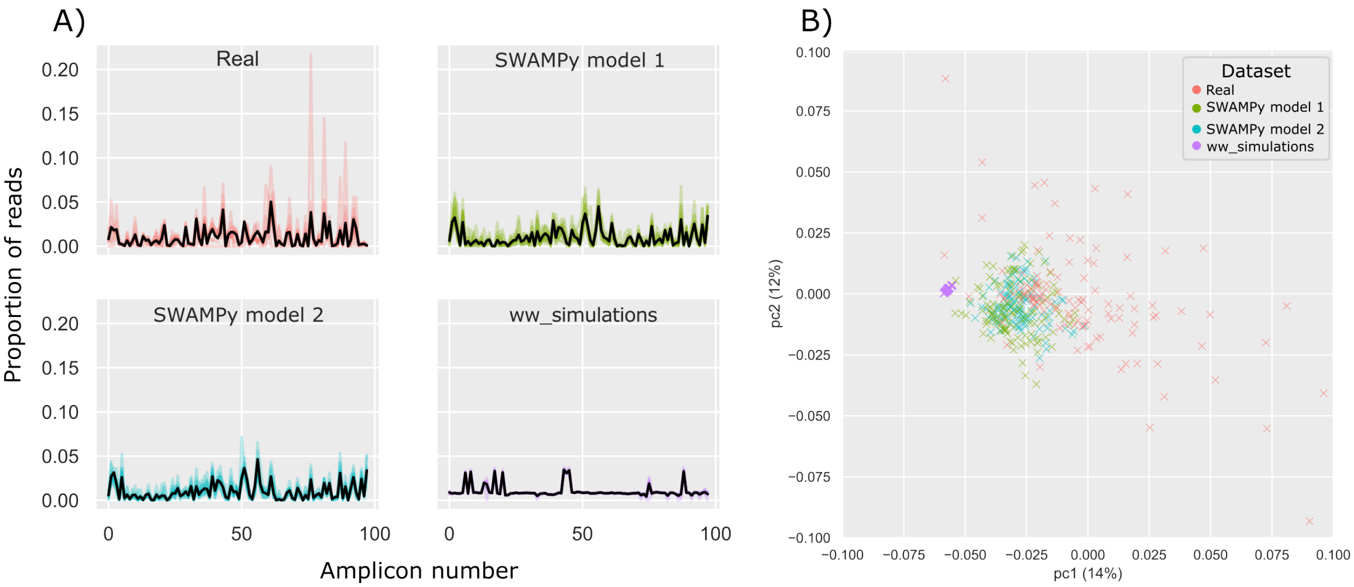

Fig. 3: **Comparison of real and simulated amplicon proportions.** A) Amplicon distribution profiles (normalised as proportions of total reads). In black we show the mean distribution profile. The coloured backgrounds are profiles from 5 randomly chosen samples. B) PCA plot showing the variability in amplicon distribution profiles across the real dataset. The two principal components together represent 26% of the variation in the real amplicon data. Amplicon distributions from the 3 simulated datasets are then projected onto these two principal components.

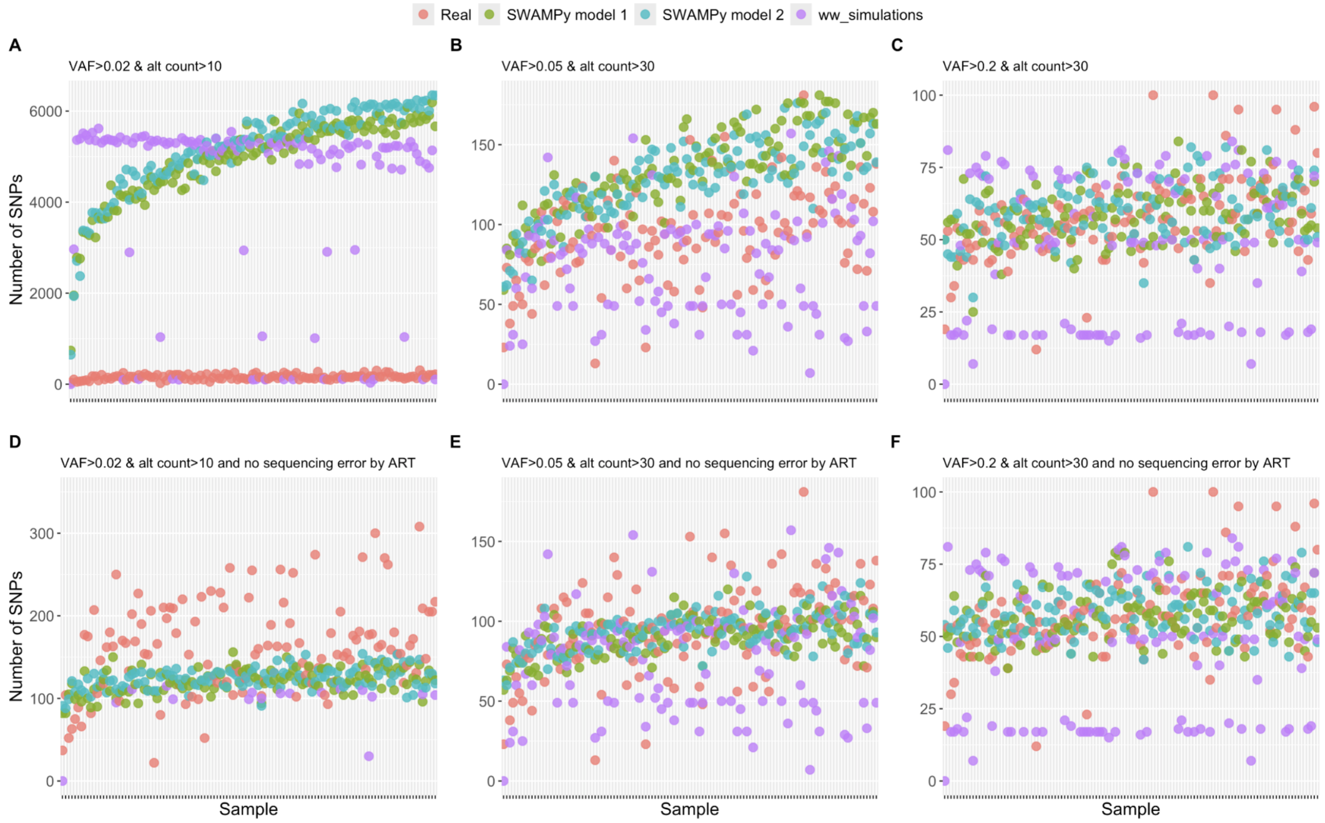

Fig. 4: **Comparison of variant sites between real data and simulations.** On the Y axis we show the number of real data and simulated SNPs under different filtering criteria. The top and bottom rows were derived with and without read errors from ART, respectively. Samples are ordered on the X axis according to total coverage in the real data. **A**,**D** SNPs with $> 2\%$ frequency and > 10 reads supporting the alternative allele. **B**,**E** SNPs with $> 5\%$ frequency and > 30 reads supporting the alternative allele. **C**,**F** SNPs with $> 20\%$ frequency and > 30 reads supporting the alternative allele.

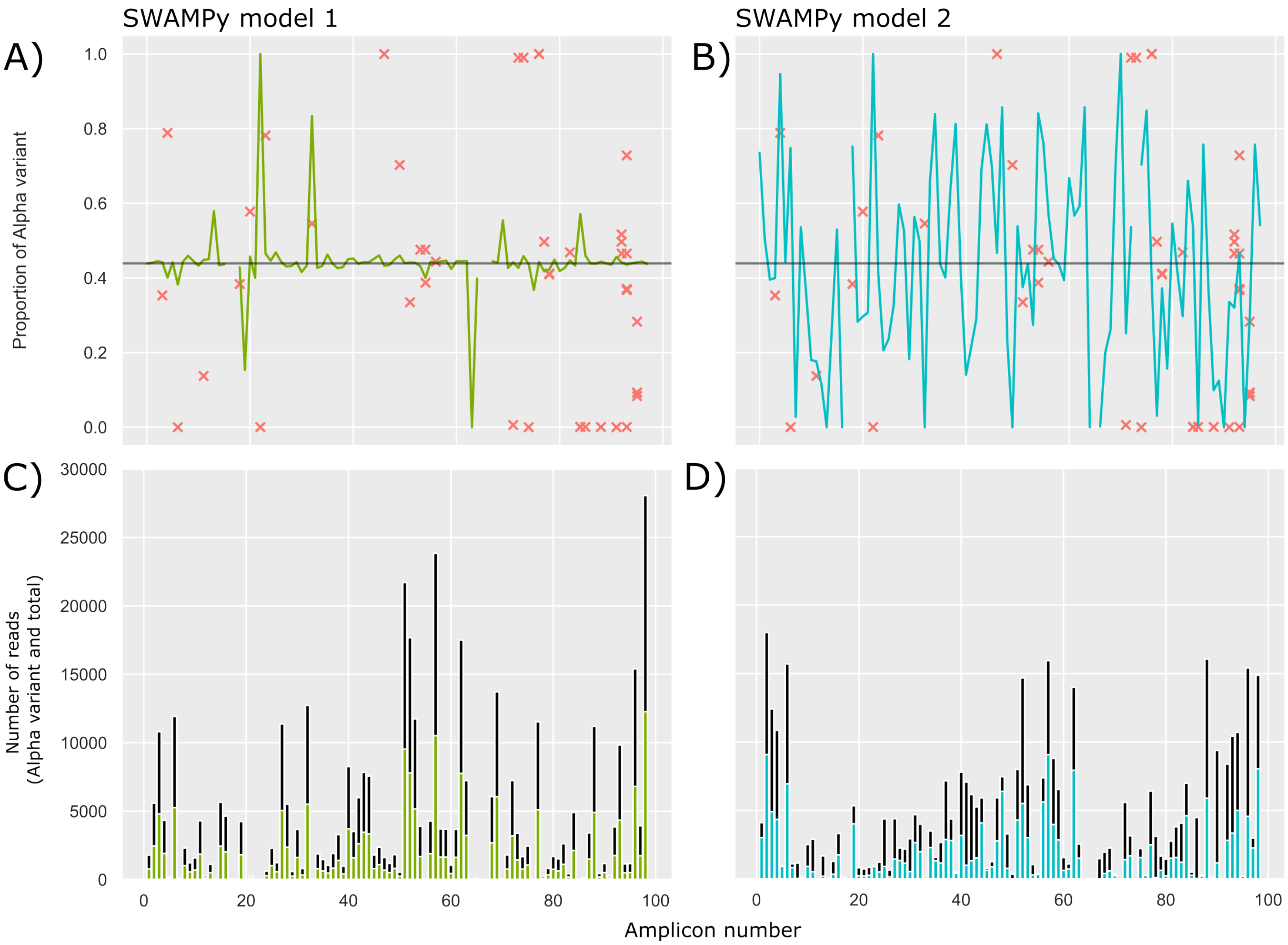

Fig. 5: **SWAMPy model 1 and 2 amplicon proportions from a case-study simulation experiment.** Simulated amplicon proportions and numbers of reads based on real sample SRR18328793, which was estimated by Freyja as 43% Alpha, 50% Epsilon, 7% other. A, B) Proportions of Alpha variant amplicons generated by SWAMPy using respectively models 1 and 2. The black horizontal line is at 43%. Red markers indicate the proportions in the real data of Alpha vs Epsilon SNPs. C, D) Amplicon read counts using respectively model 1 and 2; coloured bars indicate Alpha variant reads whereas the black bar is the total number of reads.

time	f(Alpha)	f(Delta)	f(Omicron)	time	f(Alpha)	f(Delta)	f(Omicron)
0	1000	0	0				
1	990	10	0	37	617	357	26
2	989	11	0	38	574	391	35
3	988	12	0	39	526	426	47
4	986	14	0	40	474	464	63
5	985	15	0	41	420	497	83
6	983	17	0	42	370	521	109
7	982	18	0	43	324	535	141
8	980	20	0	44	280	538	182
9	978	22	0	45	239	530	231
10	975	25	0	46	201	511	288
11	973	27	0	47	166	481	353
12	970	30	0	48	134	441	425
13	967	33	0	49	106	395	499
14	963	37	0	50	82	344	574
15	959	41	0	51	62	293	645
16	955	45	0	52	46	244	710
17	950	50	0	53	34	198	768
18	945	55	0	54	24	159	817
19	939	61	0	55	17	125	858
20	933	67	0	56	12	97	891
21	926	74	0	57	8	75	917
22	918	82	0	58	6	57	937
23	909	90	0	59	4	44	952
24	900	100	1	60	3	33	964
25	889	110	1	61	2	25	973
26	877	122	1	62	1	19	980
27	864	135	1	63	1	14	985
28	849	149	2	64	1	10	989
29	833	164	2	65	0	8	992
30	815	181	3	66	0	6	994
31	795	200	4	67	0	4	995
32	773	221	6	68	0	3	997
33	748	244	8	69	0	2	998
34	721	269	11	70	0	2	998
35	690	296	15	71	0	1	999
36	655	325	20	72	0	0	1000

Table 1. Simulated genome proportions

The non-default SWAMPy options used to simulate these 73 time points are:

```
-ins 0.0002
-del 0.00115
-subs 0.005
-rins 0.0002
-rdel 0.00115
-subs 0.005
--amplicon_distribution dirichlet_2
--amplicon_pseudocounts 200
```

References

P. Danecek, J. K. Bonfield, J. Liddle, J. Marshall, V. Ohan, M. O. Pollard, A. Whitwham, T. Keane, S. A. McCarthy, R. M. Davies, and H. Li. Twelve years of SAMtools and BCFtools. *GigaScience*, 10, Feb. 2021. doi: 10.1093/gigascience/giab008.

S. Delbue, S. D'Alessandro, L. Signorini, M. Dolci, E. Pariani, M. Bianchi, S. Fattori, A. Modenese, C. Galli, I. Eberini, and P. Ferrante. Isolation of SARS-CoV-2 strains carrying a nucleotide mutation, leading to a stop codon in the orf 6 protein. *Emerging Microbes & Infections*, 10:252–255, Feb. 2021. doi: 10.1080/22221751.2021.1884003.

W. Huang, L. Li, J. R. Myers, and G. T. Marth. ART: a next-generation sequencing read simulator. *Bioinformatics*, 28:593–594, Dec. 2011. doi: 10.1093/bioinformatics/btr708.

I. Jungreis, R. Sealfon, and M. Kellis. SARS-CoV-2 gene content and COVID-19 mutation impact by comparing 44 *Sarbecovirus* genomes. *Nature Communications*, 12:2642, May 2021. doi: 10.1038/s41467-021-22905-7.

S. Karthikeyan, J. I. Levy, P. D. Hoff, G. Humphrey, A. Birmingham, K. Jepsen, S. Farmer, H. M. Tubb, T. Valles, C. E. Tribelhorn, R. Tsai, S. Aigner, S. Sathe, N. Moshiri, B. Henson, A. M. Mark, A. Hakim, N. A. Baer, T. Barber, P. Belda-Ferre, M. Chacón, W. Cheung, E. S. Cresini, E. R. Eisner, A. L. Lastrella, E. S. Lawrence, C. A. Marotz, T. T. Ngo, T. Ostrander, A. Plascencia, R. A. Salido, P. Seaver, E. W. Smoot, D. McDonald, R. M. Neuhard, A. L. Scioscia, A. M. Satterlund, E. H. Simmons, D. B. Abelman, D. Brenner, J. C. Bruner, A. Buckley, M. Ellison, J. Gattas, S. L. Gonias, M. Hale, F. Hawkins, L. Ikeda, H. Jhaveri, T. Johnson, V. Kellen, B. Kremer, G. Matthews, R. W. McLawhon, P. Ouillet, D. Park, A. Pradenas, S. Reed, L. Riggs, A. Sanders, B. Sollenberger, A. Song, B. White, T. Winbush, C. M. Aceves, C. Anderson, K. Gangavarapu, E. Hufbauer, E. Kurzban, J. Lee, N. L. Matteson, E. Parker, S. A. Perkins, K. S. Ramesh, R. Robles-Sikisaka, M. A. Schwab, E. Spencer, S. Wohl, L. Nicholson, I. H. McHardy, D. P. Dimmock, C. A. Hobbs, O. Bakhtar, A. Harding, A. Mendoza, A. Bolze, D. Becker, E. T. Cirulli, M. Isaksson, K. M. S. Barrett, N. L. Washington, J. D. Malone, A. M. Schafer, N. Gurfield, S. Stous, R. Fielding-Miller, R. S. Garfein, T. Gaines, C. Anderson, N. K. Martin, R. Schooley, B. Austin, D. R. MacCannell, S. F. Kingsmore, W. Lee, S. Shah, E. McDonald, A. T. Yu, M. Zeller, K. M. Fisch, C. Longhurst, P. Maysent, D. Pride, P. K. Khosla, L. C. Laurent, G. W. Yeo, K. G. Andersen, and R. Knight. Wastewater sequencing reveals early cryptic SARS-CoV-2 variant transmission. *Nature*, 609: 101–108, Sep. 2022. doi: 10.1038/s41586-022-05049-6.

K. Lange. Applications of the Dirichlet distribution to forensic match probabilities. *Genetica*, 96:107–117, Jun. 1995. doi: 10.1007/BF01441156.

B. Langmead and S. L. Salzberg. Fast gapped-read alignment with Bowtie 2. *Nature Methods*, 9:357–359, Apr. 2012. doi: 10.1038/nmeth.1923.

A. Rambaut, E. C. Holmes, A. O'Toole, V. Hill, J. T. McCrone, C. Ruis, L. du Plessis, and O. G. Pybus. A dynamic nomenclature proposal for sars-cov-2 lineages to assist genomic epidemiology. *Nature Microbiology*, 5:1403, Nov. 2020. doi: 10.1038/s41564-020-0770-5.

N. Stoler and A. Nekrutenko. Sequencing error profiles of Illumina sequencing instruments. *NAR Genomics and Bioinformatics*, 3: lqab019, Mar. 2021. doi: 10.1093/nargab/lqab019.

F. Wu, S. Zhao, B. Yu, Y. M. Chen, W. Wang, Z. G. Song, Y. Hu, Z. W. Tao, J. H. Tian, Y. Y. Pei, M. L. Yuan, Y. L. Zhang, F. H. Dai, Y. Liu, Q. M. Wang, J. J. Zheng, L. Xu, E. C. Holmes, and Y. Z. Zhang. A new coronavirus associated with human respiratory disease in China. *Nature*, 579:265, Mar. 2020. doi: 10.1038/S41586-020-2008-3.
